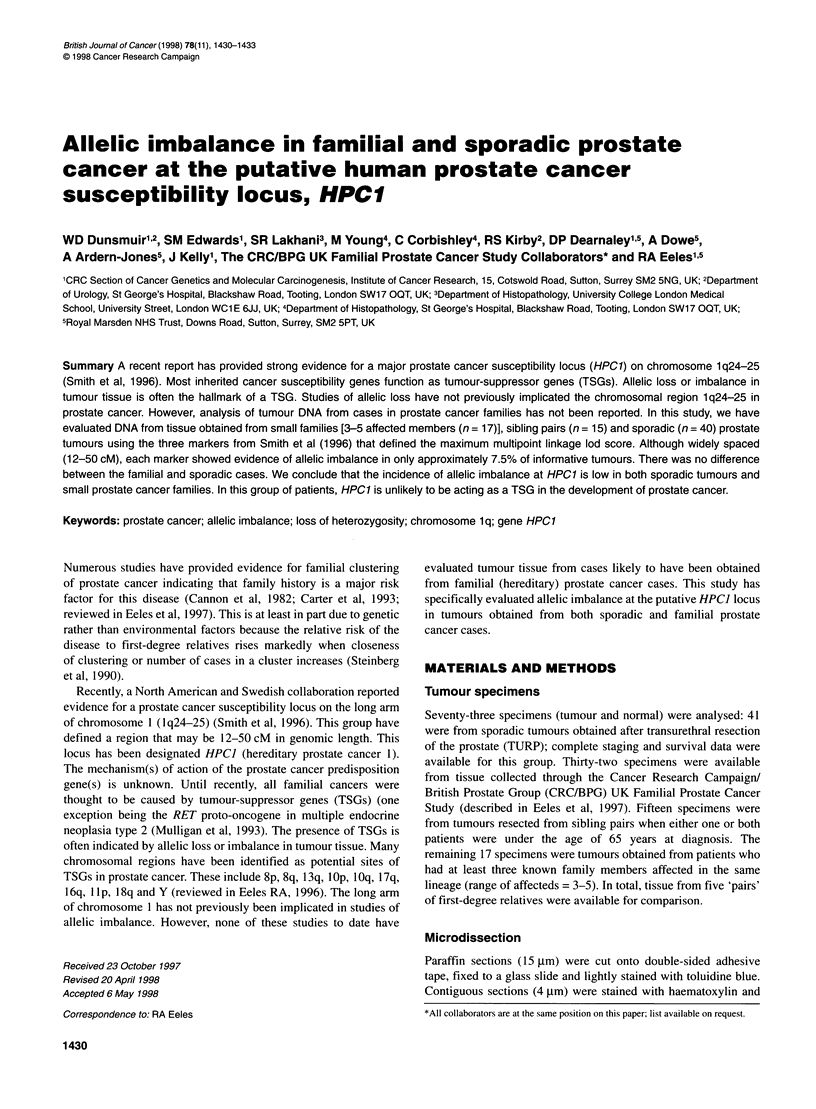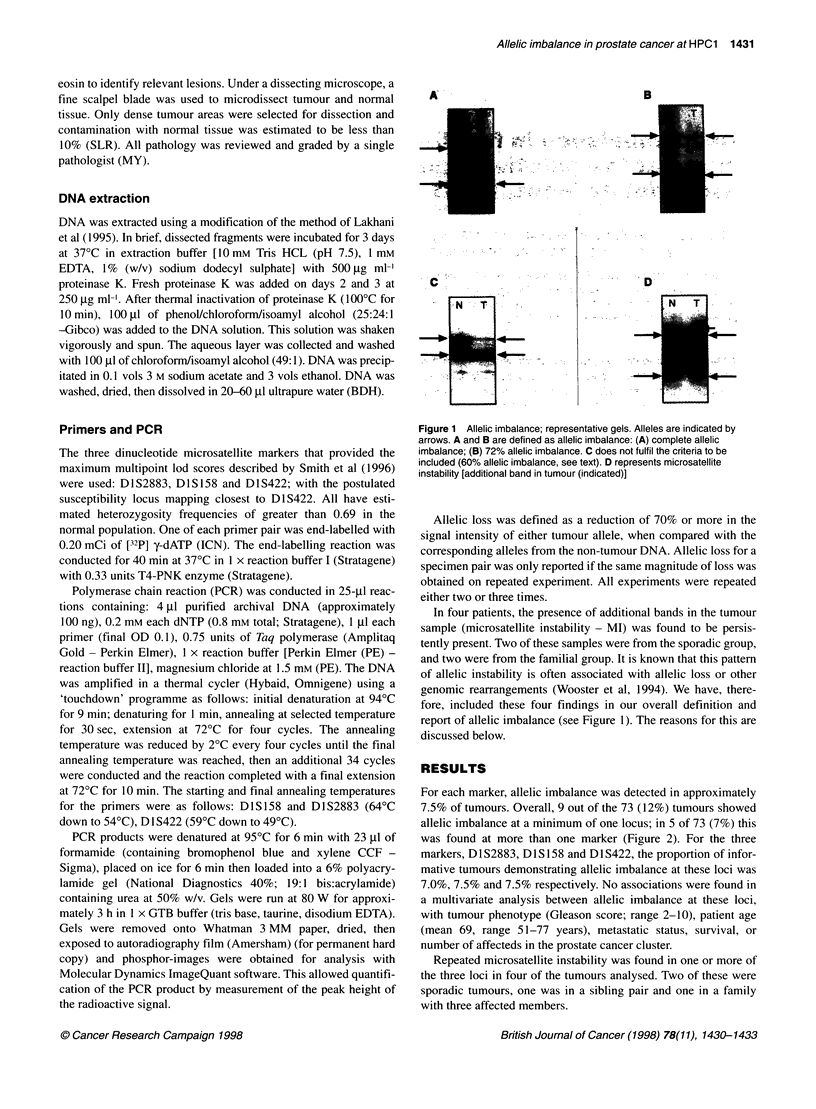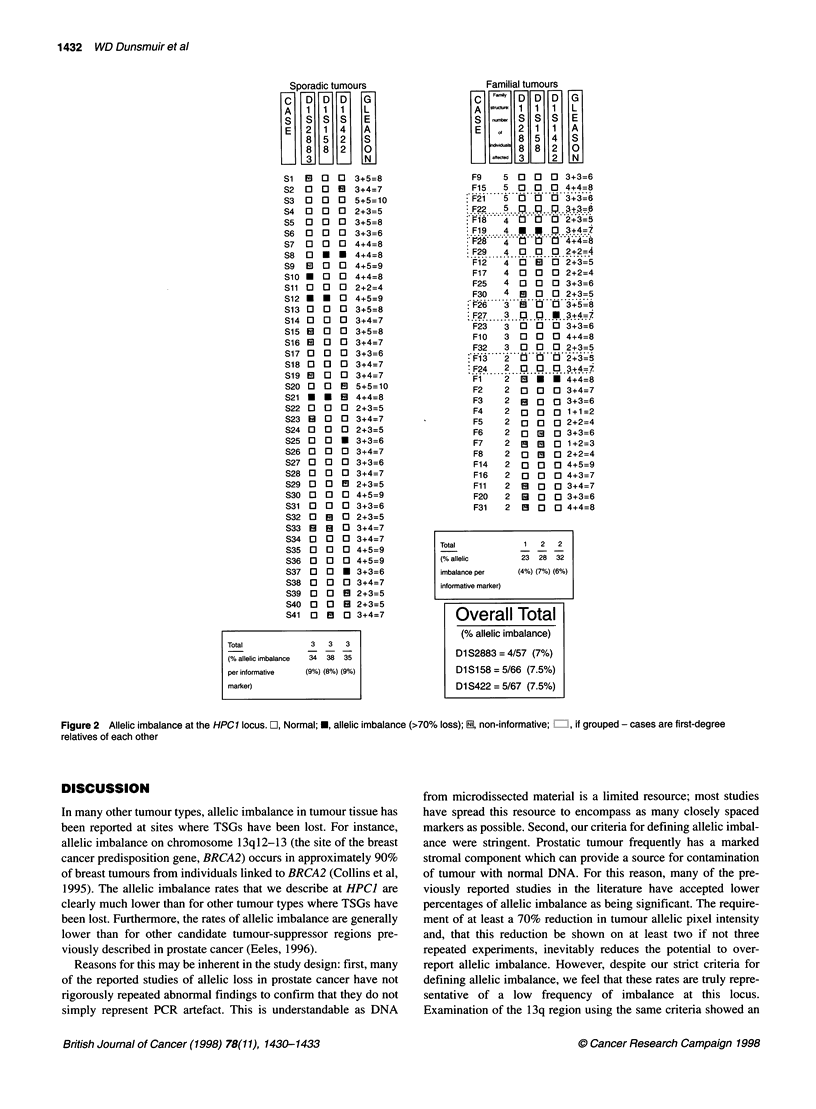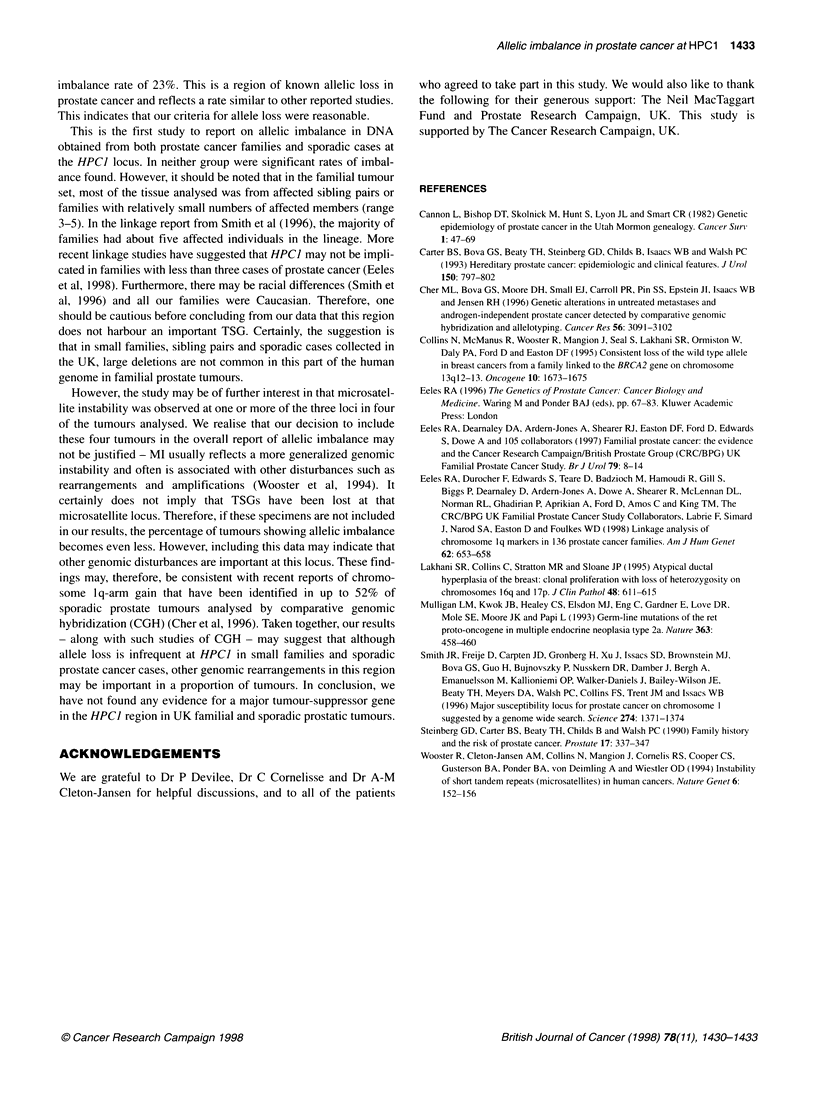# Allelic imbalance in familial and sporadic prostate cancer at the putative human prostate cancer susceptibility locus, HPC1. CRC/BPG UK Familial Prostate Cancer Study Collaborators. Cancer Research Campaign/British Prostate Group.

**DOI:** 10.1038/bjc.1998.703

**Published:** 1998-12

**Authors:** W. D. Dunsmuir, S. M. Edwards, S. R. Lakhani, M. Young, C. Corbishley, R. S. Kirby, D. P. Dearnaley, A. Dowe, A. Ardern-Jones, J. Kelly, R. A. Eeles

**Affiliations:** CRC Section of Cancer Genetics and Molecular Carcinogenesis, Institute of Cancer Research, Sutton, Surrey, UK.

## Abstract

**Images:**


British Journal of Cancer (1998) 78(11), 1430–1433
© 1998 Cancer Research Campaign

### Allelic imbalance in familial and sporadic prostate cancer at the putative human prostate cancer susceptibility locus, *HPC1*

**WD Dunsmuir[1,2], SM Edwards[1], SR Lakhani[3], M Young[4], C Corbishley[4], RS Kirby[2], DP Dearnaley[1,5], A Dowe[5], A Ardern-Jones[5], J Kelly[1], The CRC/BPG UK Familial Prostate Cancer Study Collaborators* and RA Eeles[1,5]**

[1]CRC Section of Cancer Genetics and Molecular Carcinogenesis, Institute of Cancer Research, 15, Cotswold Road, Sutton, Surrey SM2 5NG, UK; [2]Department of Urology, St George's Hospital, Blackshaw Road, Tooting, London SW17 OQT, UK; [3]Department of Histopathology, University College London Medical School, University Street, London WC1E 6JJ, UK; [4]Department of Histopathology, St George's Hospital, Blackshaw Road, Tooting, London SW17 OQT, UK; [5]Royal Marsden NHS Trust, Downs Road, Sutton, Surrey, SM2 5PT, UK

**Summary** A recent report has provided strong evidence for a major prostate cancer susceptibility locus (*HPC1*) on chromosome 1q24–25 (Smith et al, 1996). Most inherited cancer susceptibility genes function as tumour-suppressor genes (TSGs). Allelic loss or imbalance in tumour tissue is often the hallmark of a TSG. Studies of allelic loss have not previously implicated the chromosomal region 1q24–25 in prostate cancer. However, analysis of tumour DNA from cases in prostate cancer families has not been reported. In this study, we have evaluated DNA from tissue obtained from small families [3–5 affected members ($n = 17$)], sibling pairs ($n = 15$) and sporadic ($n = 40$) prostate tumours using the three markers from Smith et al (1996) that defined the maximum multipoint linkage lod score. Although widely spaced (12–50 cM), each marker showed evidence of allelic imbalance in only approximately 7.5% of informative tumours. There was no difference between the familial and sporadic cases. We conclude that the incidence of allelic imbalance at *HPC1* is low in both sporadic tumours and small prostate cancer families. In this group of patients, *HPC1* is unlikely to be acting as a TSG in the development of prostate cancer.

**Keywords:** prostate cancer; allelic imbalance; loss of heterozygosity; chromosome 1q; gene *HPC1*

Numerous studies have provided evidence for familial clustering of prostate cancer indicating that family history is a major risk factor for this disease (Cannon et al, 1982; Carter et al, 1993; reviewed in Eeles et al, 1997). This is at least in part due to genetic rather than environmental factors because the relative risk of the disease to first-degree relatives rises markedly when closeness of clustering or number of cases in a cluster increases (Steinberg et al, 1990).

Recently, a North American and Swedish collaboration reported evidence for a prostate cancer susceptibility locus on the long arm of chromosome 1 (1q24–25) (Smith et al, 1996). This group have defined a region that may be 12–50 cM in genomic length. This locus has been designated *HPC1* (hereditary prostate cancer 1). The mechanism(s) of action of the prostate cancer predisposition gene(s) is unknown. Until recently, all familial cancers were thought to be caused by tumour-suppressor genes (TSGs) (one exception being the *RET* proto-oncogene in multiple endocrine neoplasia type 2 (Mulligan et al, 1993). The presence of TSGs is often indicated by allelic loss or imbalance in tumour tissue. Many chromosomal regions have been identified as potential sites of TSGs in prostate cancer. These include 8p, 8q, 13q, 10p, 10q, 17q, 16q, 11p, 18q and Y (reviewed in Eeles RA, 1996). The long arm of chromosome 1 has not previously been implicated in studies of allelic imbalance. However, none of these studies to date have evaluated tumour tissue from cases likely to have been obtained from familial (hereditary) prostate cancer cases. This study has specifically evaluated allelic imbalance at the putative *HPC1* locus in tumours obtained from both sporadic and familial prostate cancer cases.

*Received 23 October 1997*
*Revised 20 April 1998*
*Accepted 6 May 1998*

*Correspondence to:* RA Eeles

### MATERIALS AND METHODS

### Tumour specimens

Seventy-three specimens (tumour and normal) were analysed: 41 were from sporadic tumours obtained after transurethral resection of the prostate (TURP); complete staging and survival data were available for this group. Thirty-two specimens were available from tissue collected through the Cancer Research Campaign/ British Prostate Group (CRC/BPG) UK Familial Prostate Cancer Study (described in Eeles et al, 1997). Fifteen specimens were from tumours resected from sibling pairs when either one or both patients were under the age of 65 years at diagnosis. The remaining 17 specimens were tumours obtained from patients who had at least three known family members affected in the same lineage (range of affecteds = 3–5). In total, tissue from five 'pairs' of first-degree relatives were available for comparison.

### Microdissection

Paraffin sections (15 μm) were cut onto double-sided adhesive tape, fixed to a glass slide and lightly stained with toluidine blue. Contiguous sections (4 μm) were stained with haematoxylin and

*All collaborators are at the same position on this paper; list available on request.

eosin to identify relevant lesions. Under a dissecting microscope, a fine scalpel blade was used to microdissect tumour and normal tissue. Only dense tumour areas were selected for dissection and contamination with normal tissue was estimated to be less than 10% (SLR). All pathology was reviewed and graded by a single pathologist (MY).

### DNA extraction

DNA was extracted using a modification of the method of Lakhani et al (1995). In brief, dissected fragments were incubated for 3 days at 37°C in extraction buffer [10 mM Tris HCL (pH 7.5), 1 mM EDTA, 1% (w/v) sodium dodecyl sulphate] with 500 µg ml$^{-1}$ proteinase K. Fresh proteinase K was added on days 2 and 3 at 250 µg ml$^{-1}$. After thermal inactivation of proteinase K (100°C for 10 min), 100 µl of phenol/chloroform/isoamyl alcohol (25:24:1 –Gibco) was added to the DNA solution. This solution was shaken vigorously and spun. The aqueous layer was collected and washed with 100 µl of chloroform/isoamyl alcohol (49:1). DNA was precipitated in 0.1 vols 3 M sodium acetate and 3 vols ethanol. DNA was washed, dried, then dissolved in 20–60 µl ultrapure water (BDH).

### Primers and PCR

The three dinucleotide microsatellite markers that provided the maximum multipoint lod scores described by Smith et al (1996) were used: D1S2883, D1S158 and D1S422; with the postulated susceptibility locus mapping closest to D1S422. All have estimated heterozygosity frequencies of greater than 0.69 in the normal population. One of each primer pair was end-labelled with 0.20 mCi of [$^{32}$P] γ-dATP (ICN). The end-labelling reaction was conducted for 40 min at 37°C in 1 × reaction buffer I (Stratagene) with 0.33 units T4-PNK enzyme (Stratagene).

Polymerase chain reaction (PCR) was conducted in 25-µl reactions containing: 4 µl purified archival DNA (approximately 100 ng), 0.2 mM each dNTP (0.8 mM total; Stratagene), 1 µl each primer (final OD 0.1), 0.75 units of *Taq* polymerase (Amplitaq Gold – Perkin Elmer), 1 × reaction buffer [Perkin Elmer (PE) – reaction buffer II], magnesium chloride at 1.5 mM (PE). The DNA was amplified in a thermal cycler (Hybaid, Omnigene) using a 'touchdown' programme as follows: initial denaturation at 94°C for 9 min; denaturing for 1 min, annealing at selected temperature for 30 sec, extension at 72°C for four cycles. The annealing temperature was reduced by 2°C every four cycles until the final annealing temperature was reached, then an additional 34 cycles were conducted and the reaction completed with a final extension at 72°C for 10 min. The starting and final annealing temperatures for the primers were as follows: D1S158 and D1S2883 (64°C down to 54°C), D1S422 (59°C down to 49°C).

PCR products were denatured at 95°C for 6 min with 23 µl of formamide (containing bromophenol blue and xylene CCF – Sigma), placed on ice for 6 min then loaded into a 6% polyacrylamide gel (National Diagnostics 40%; 19:1 bis:acrylamide) containing urea at 50% w/v. Gels were run at 80 W for approximately 3 h in 1 × GTB buffer (tris base, taurine, disodium EDTA). Gels were removed onto Whatman 3 MM paper, dried, then exposed to autoradiography film (Amersham) (for permanent hard copy) and phosphor-images were obtained for analysis with Molecular Dynamics ImageQuant software. This allowed quantification of the PCR product by measurement of the peak height of the radioactive signal.

**Figure 1** Allelic imbalance; representative gels. Alleles are indicated by arrows. **A** and **B** are defined as allelic imbalance: (**A**) complete allelic imbalance; (**B**) 72% allelic imbalance. **C** does not fulfil the criteria to be included (60% allelic imbalance, see text). **D** represents microsatellite instability [additional band in tumour (indicated)]

Allelic loss was defined as a reduction of 70% or more in the signal intensity of either tumour allele, when compared with the corresponding alleles from the non-tumour DNA. Allelic loss for a specimen pair was only reported if the same magnitude of loss was obtained on repeated experiment. All experiments were repeated either two or three times.

In four patients, the presence of additional bands in the tumour sample (microsatellite instability – MI) was found to be persistently present. Two of these samples were from the sporadic group, and two were from the familial group. It is known that this pattern of allelic instability is often associated with allelic loss or other genomic rearrangements (Wooster et al, 1994). We have, therefore, included these four findings in our overall definition and report of allelic imbalance (see Figure 1). The reasons for this are discussed below.

### RESULTS

For each marker, allelic imbalance was detected in approximately 7.5% of tumours. Overall, 9 out of the 73 (12%) tumours showed allelic imbalance at a minimum of one locus; in 5 of 73 (7%) this was found at more than one marker (Figure 2). For the three markers, D1S2883, D1S158 and D1S422, the proportion of informative tumours demonstrating allelic imbalance at these loci was 7.0%, 7.5% and 7.5% respectively. No associations were found in a multivariate analysis between allelic imbalance at these loci, with tumour phenotype (Gleason score; range 2–10), patient age (mean 69, range 51–77 years), metastatic status, survival, or number of affecteds in the prostate cancer cluster.

Repeated microsatellite instability was found in one or more of the three loci in four of the tumours analysed. Two of these were sporadic tumours, one was in a sibling pair and one in a family with three affected members.

© Cancer Research Campaign 1998

**Figure 2** Allelic imbalance at the *HPC1* locus. □, Normal; ■, allelic imbalance (>70% loss); NI, non-informative; ⬚, if grouped – cases are first-degree relatives of each other

### DISCUSSION

In many other tumour types, allelic imbalance in tumour tissue has been reported at sites where TSGs have been lost. For instance, allelic imbalance on chromosome 13q12–13 (the site of the breast cancer predisposition gene, *BRCA2*) occurs in approximately 90% of breast tumours from individuals linked to *BRCA2* (Collins et al, 1995). The allelic imbalance rates that we describe at *HPC1* are clearly much lower than for other tumour types where TSGs have been lost. Furthermore, the rates of allelic imbalance are generally lower than for other candidate tumour-suppressor regions previously described in prostate cancer (Eeles, 1996).

Reasons for this may be inherent in the study design: first, many of the reported studies of allelic loss in prostate cancer have not rigorously repeated abnormal findings to confirm that they do not simply represent PCR artefact. This is understandable as DNA from microdissected material is a limited resource; most studies have spread this resource to encompass as many closely spaced markers as possible. Second, our criteria for defining allelic imbalance were stringent. Prostatic tumour frequently has a marked stromal component which can provide a source for contamination of tumour with normal DNA. For this reason, many of the previously reported studies in the literature have accepted lower percentages of allelic imbalance as being significant. The requirement of at least a 70% reduction in tumour allelic pixel intensity and, that this reduction be shown on at least two if not three repeated experiments, inevitably reduces the potential to over-report allelic imbalance. However, despite our strict criteria for defining allelic imbalance, we feel that these rates are truly representative of a low frequency of imbalance at this locus. Examination of the 13q region using the same criteria showed an

© Cancer Research Campaign 1998

imbalance rate of 23%. This is a region of known allelic loss in prostate cancer and reflects a rate similar to other reported studies. This indicates that our criteria for allele loss were reasonable.

This is the first study to report on allelic imbalance in DNA obtained from both prostate cancer families and sporadic cases at the *HPC1* locus. In neither group were significant rates of imbalance found. However, it should be noted that in the familial tumour set, most of the tissue analysed was from affected sibling pairs or families with relatively small numbers of affected members (range 3–5). In the linkage report from Smith et al (1996), the majority of families had about five affected individuals in the lineage. More recent linkage studies have suggested that *HPC1* may not be implicated in families with less than three cases of prostate cancer (Eeles et al, 1998). Furthermore, there may be racial differences (Smith et al, 1996) and all our families were Caucasian. Therefore, one should be cautious before concluding from our data that this region does not harbour an important TSG. Certainly, the suggestion is that in small families, sibling pairs and sporadic cases collected in the UK, large deletions are not common in this part of the human genome in familial prostate tumours.

However, the study may be of further interest in that microsatellite instability was observed at one or more of the three loci in four of the tumours analysed. We realise that our decision to include these four tumours in the overall report of allelic imbalance may not be justified – MI usually reflects a more generalized genomic instability and often is associated with other disturbances such as rearrangements and amplifications (Wooster et al, 1994). It certainly does not imply that TSGs have been lost at that microsatellite locus. Therefore, if these specimens are not included in our results, the percentage of tumours showing allelic imbalance becomes even less. However, including this data may indicate that other genomic disturbances are important at this locus. These findings may, therefore, be consistent with recent reports of chromosome 1q-arm gain that have been identified in up to 52% of sporadic prostate tumours analysed by comparative genomic hybridization (CGH) (Cher et al, 1996). Taken together, our results – along with such studies of CGH – may suggest that although allele loss is infrequent at *HPC1* in small families and sporadic prostate cancer cases, other genomic rearrangements in this region may be important in a proportion of tumours. In conclusion, we have not found any evidence for a major tumour-suppressor gene in the *HPC1* region in UK familial and sporadic prostatic tumours.

### ACKNOWLEDGEMENTS

We are grateful to Dr P Devilee, Dr C Cornelisse and Dr A-M Cleton-Jansen for helpful discussions, and to all of the patients who agreed to take part in this study. We would also like to thank the following for their generous support: The Neil MacTaggart Fund and Prostate Research Campaign, UK. This study is supported by The Cancer Research Campaign, UK.

### REFERENCES

Cannon L, Bishop DT, Skolnick M, Hunt S, Lyon JL and Smart CR (1982) Genetic epidemiology of prostate cancer in the Utah Mormon genealogy. *Cancer Surv* **1**: 47–69

Carter BS, Bova GS, Beaty TH, Steinberg GD, Childs B, Isaacs WB and Walsh PC (1993) Hereditary prostate cancer: epidemiologic and clinical features. *J Urol* **150**: 797–802

Cher ML, Bova GS, Moore DH, Small EJ, Carroll PR, Pin SS, Epstein JI, Isaacs WB and Jensen RH (1996) Genetic alterations in untreated metastases and androgen-independent prostate cancer detected by comparative genomic hybridization and allelotyping. *Cancer Res* **56**: 3091–3102

Collins N, McManus R, Wooster R, Mangion J, Seal S, Lakhani SR, Ormiston W, Daly PA, Ford D and Easton DF (1995) Consistent loss of the wild type allele in breast cancers from a family linked to the *BRCA2* gene on chromosome 13q12–13. *Oncogene* **10**: 1673–1675

Eeles RA (1996) *The Genetics of Prostate Cancer: Cancer Biology and Medicine*. Waring M and Ponder BAJ (eds), pp. 67–83. Kluwer Academic Press: London

Eeles RA, Dearnaley DA, Ardern-Jones A, Shearer RJ, Easton DF, Ford D, Edwards S, Dowe A and 105 collaborators (1997) Familial prostate cancer: the evidence and the Cancer Research Campaign/British Prostate Group (CRC/BPG) UK Familial Prostate Cancer Study. *Br J Urol* **79**: 8–14

Eeles RA, Durocher F, Edwards S, Teare D, Badzioch M, Hamoudi R, Gill S, Biggs P, Dearnaley D, Ardern-Jones A, Dowe A, Shearer R, McLennan DL, Norman RL, Ghadirian P, Aprikian A, Ford D, Amos C and King TM, The CRC/BPG UK Familial Prostate Cancer Study Collaborators, Labrie F, Simard J, Narod SA, Easton D and Foulkes WD (1998) Linkage analysis of chromosome 1q markers in 136 prostate cancer families. *Am J Hum Genet* **62**: 653–658

Lakhani SR, Collins C, Stratton MR and Sloane JP (1995) Atypical ductal hyperplasia of the breast: clonal proliferation with loss of heterozygosity on chromosomes 16q and 17p. *J Clin Pathol* **48**: 611–615

Mulligan LM, Kwok JB, Healey CS, Elsdon MJ, Eng C, Gardner E, Love DR, Mole SE, Moore JK and Papi L (1993) Germ-line mutations of the ret proto-oncogene in multiple endocrine neoplasia type 2a. *Nature* **363**: 458–460

Smith JR, Freije D, Carpten JD, Gronberg H, Xu J, Issacs SD, Brownstein MJ, Bova GS, Guo H, Bujnovszky P, Nusskern DR, Damber J, Bergh A, Emanuelsson M, Kallioniemi OP, Walker-Daniels J, Bailey-Wilson JE, Beaty TH, Meyers DA, Walsh PC, Collins FS, Trent JM and Issacs WB (1996) Major susceptibility locus for prostate cancer on chromosome 1 suggested by a genome wide search. *Science* **274**: 1371–1374

Steinberg GD, Carter BS, Beaty TH, Childs B and Walsh PC (1990) Family history and the risk of prostate cancer. *Prostate* **17**: 337–347

Wooster R, Cleton-Jansen AM, Collins N, Mangion J, Cornelis RS, Cooper CS, Gusterson BA, Ponder BA, von Deimling A and Wiestler OD (1994) Instability of short tandem repeats (microsatellites) in human cancers. *Nature Genet* **6**: 152–156

© Cancer Research Campaign 1998